# Supplementary material for: Diagnostic test accuracy of artificial intelligence in screening for referable diabetic retinopathy in real-world settings: A systematic review and meta-analysis
Source: PLOS Glob Public Health. 2023 Sep 20;3(9):e0002160. doi: 10.1371/journal.pgph.0002160 (PMC10511145; doi:10.1371/journal.pgph.0002160)
Supplement: S1 Table — (DOCX) [file pgph.0002160.s001.docx]

All searches were done on February 9, 2023, without any restrictions on language and year of publication.

**1. Medical Literature Analysis and Retrieval System Online (MEDLINE) via PubMed**

**Table 1.** **Search strategy in our study on the electronic database, PubMed.**

| **Target Condition** | | |
| --- | --- | --- |
| #1 | MeSH | Diabetic Retinopathy |
| #2 | Text Word | Diabetic Retinopathy OR Diabetic Eye Disease OR Referable DR |
| #3 | #1 OR #2 | |
| **Intervention** | | |
| #4 | MeSH | Artificial Intelligence OR Machine Learning OR Deep Learning OR Convolutional Neural Network |
| #5 | MeSH | Diabetic Retinopathy Screening OR Eye Screening |
| #6 | Text Word | Artificial Intelligence OR Machine Learning OR Deep Learning OR Convolutional Neural Network OR AI OR Screening OR DR Screening |
| #7 | #4 OR #5 OR #6 | |
| **Outcome** | | |
| #8 | MeSH | Diagnostic Accuracy |
| #9 | Text Word | Diagnostic Accuracy OR Sensitivity OR Specificity OR AUC |
| #10 | #8 OR #9 | |
| #11 | #3 AND #7 AND #10 | |

**2. Cochrane Central Register of Controlled Trials (CENTRAL)**

**Table 2.** **Search strategy in our study on the electronic database, CENTRAL.**

| **Target Condition** | | |
| --- | --- | --- |
| #1 | MeSH | [Diabetic Retinopathy] explode all trees |
| #2 | All Text | (Diabetic Retinopathy OR Diabetic Eye Disease OR Referable DR) |
| #3 | #1 OR #2 | |
| **Intervention** | | |
| #4 | MeSH | [Artificial Intelligence] explode all trees |
| #5 | MeSH | [Machine Learning] explode all trees |
| #6 | MeSH | [Deep Learning] explode all trees |
| #7 | #4 OR #5 OR #6 | |
| #8 | Artificial Intelligence OR Machine Learning OR Deep Learning OR Convolutional Neural Network | |
| #9 | #7 OR #8 | |
| #10 | MeSH | [Diagnostic Screening Programs] explode all trees |
| #11 | All Text | (Diabetic Retinopathy Screening OR Eye Screening) |
| #12 | #10 OR #11 | |
| #13 | #9 OR #12 | |
| **Outcome** | | |
| #14 | MeSH | [Dimensional Measurement Accuracy] explode all trees |
| #15 | Text Word | (Diagnostic Accuracy OR Sensitivity OR Specificity OR AUC) |
| #16 | #14 OR #15 | |
| #17 | #3 AND #9 AND #13 AND #16 | |

**3. Cumulative Index to Nursing and Allied Health Literature (CINAHL) through EBSCOhost**

**Table 3.** **Search strategy in our study on the electronic database, CINAHL.**

| **Target Condition** | | |
| --- | --- | --- |
| #1 | All Text | Diabetic Retinopathy OR Diabetic Eye Disease OR Referable DR |
| **Intervention** | | |
| #2 | All Text | Artificial Intelligence OR Machine Learning OR Deep Learning OR Convolutional Neural Network OR AI OR Screening OR DR Screening |
| **Outcome** | | |
| #3 | All Text | Diagnostic Accuracy OR Sensitivity OR Specificity OR AUC |
| #1 AND #2 AND #3 | | |

**4. Scopus**

**Table 4.** **Search strategy in our study on the electronic database, Scopus.**

| **Target Condition** | | |
| --- | --- | --- |
| #1 | All Text | Diabetic Retinopathy OR Diabetic Eye Disease OR Referable DR |
| **Intervention** | | |
| #2 | All Text | Artificial Intelligence OR Machine Learning OR Deep Learning OR Convolutional Neural Network OR AI OR Screening OR DR Screening |
| **Outcome** | | |
| #3 | All Text | Diagnostic Accuracy OR Sensitivity OR Specificity OR AUC |
| #1 AND #2 AND #3 | | |

**5. Web of Science**

**Table 5.** **Search strategy in our study on the electronic database, Web of Science.**

| **Target Condition** | | |
| --- | --- | --- |
| #1 | All Fields | Diabetic Retinopathy OR Diabetic Eye Disease OR Referable DR |
| **Intervention** | | |
| #2 | All Fields | Artificial Intelligence OR Machine Learning OR Deep Learning OR Convolutional Neural Network OR AI OR Screening OR DR Screening |
| **Outcome** | | |
| #3 | All Fields | Diagnostic Accuracy OR Sensitivity OR Specificity OR AUC |
| #1 AND #2 AND #3 | | |
