# Supplementary material for: Diagnostic test accuracy of artificial intelligence in screening for referable diabetic retinopathy in real-world settings: A systematic review and meta-analysis
Source: PLOS Glob Public Health. 2023 Sep 20;3(9):e0002160. doi: 10.1371/journal.pgph.0002160 (PMC10511145; doi:10.1371/journal.pgph.0002160)
Supplement: Attachment — Submitted filename: Response to Reviewers.pdf [file pgph.0002160.s004.pdf]

Department of Medicine
Faculty of Health Sciences
University of Cape Town
OBSERVATORY 7925
Mark E Engel, MPH PhD
J46.43 OMB GSH
E-mail: mark.engel@uct.ac.za

Segun Fatumo, PhD
Academic Editor
PLOS Global Public Health

1 August 2023

Dear Dr Segun Fatumo,

RESPONSE TO REVIEWERS (REBUTTAL LETTER)

Good day!

On behalf of our co-authors, we, the corresponding authors, thank you for offering us the opportunity to revise our manuscript entitled **Diagnostic test accuracy of artificial intelligence in screening for referable diabetic retinopathy in real-world settings: A systematic review and meta-analysis** for publication in PLOS Global Public Health. We are writing this rebuttal letter in response to each point raised by the editor and reviewers *(Please see Table 1)*.

We trust that we have adequately addressed the reviewers' concerns and that the manuscript will now be deemed acceptable for publication. Thank you for considering this work which we believe will appeal to a broad readership from research and clinical backgrounds.

Respectfully,

Mark E. Engel, MPH, PhD
South African Medical Research Council, South Africa
Department of Medicine, University of Cape Town
mark.engel@uct.ac.za

Holijah T. Uy, MD, Research fellow
Community Eye Health Institute, Faculty of Health Sciences, University of Cape Town, South Africa
uyxhol001@myuct.ac.za

"Our Mission is to be an outstanding teaching and research university, educating for life and addressing the challenges facing our society."

Table 1. Response to editor and reviewers.

Page/ Line No.	Comments from editor/reviewers	Rebuttal	Now on Page/ Line No. of Revised Manuscript (w/ track changes)
**Abstract**			
Page 2	The abstract is well-written and provides information about the study's methods, results, and interpretation. It presents the aim, methodology, key findings, and conclusions succinctly.	Thank you for the compliment. Much appreciated.	
Page 2 *Lines 42-43*	However, here are a few suggestions to improve the clarity and readability:<br><br>Be more specific with terms like 'moderate increase' and 'minimal decrease'. If possible, provide exact numbers or percentages to give the reader a clear understanding of the changes.	Added in parenthesis the sensitivities and specificities (95% CI) of the diagnostic accuracies of AI conducted in the primary-level and tertiary-level healthcare settings for comparability.	Page 2 *Lines 46-49*
Page 2 *Lines 45-46*	The conclusion might benefit from more explicit statements about the implications of the findings. You've mentioned that AI is effective in screening for RDR, but you could discuss how this impacts current practice or what the next steps could be.	Added the sentence: *it can help strengthen existing guidelines to improve current practices, especially in the LMICs.*	Page 2 *Line 52*
Page 2 *Line 35*	Line 35: The abbreviation SROC, did you want to write HSROC?	These terms differ as the SROC refers to the curve/plot, and the HSROC refers to the model used.	Page 2 *Line 38*
**Introduction**			
Page 3	The introduction is detailed and provides a good overview of the topic, clearly establishing the importance and urgency of the research. The problem is well-defined, and the literature gap that this study intends to address is well-articulated.	Thank you for the compliment.	
Page 3	However, a few areas could be improved:<br><br>A brief historical context could help to put this research's importance into perspective. For instance, what has been	Added the historical context on how DR screening is conventionally done.	Page 3 *Lines 74-79*

	the trajectory of DR treatments? What has been the impact of AI in healthcare generally?	Added the context on why AI is needed despite teleophthalmology is already available; thus, the need for AI for its impact functioning in autonomous mode.	Page 4<br>*Lines 87-91*
Page 4<br>*Lines 80-81*	Line 80-81: Ensure all the information presented has appropriate and recent references.<br><br>For example, the statement "In recent years, retrospective validation studies have shown AI to have high diagnostic accuracy in detecting DR..." needs a reference citation.	Added two recent references [11,12] for the statement, *"In recent years, retrospective validation studies have shown AI to have high diagnostic accuracy in detecting DR"*.	Page 4<br>*Line 102*
**Methods**			
Page 4	The Methods section of the systematic review appears to be largely comprehensive and transparent in its description of the processes and criteria used for the review. It carefully outlines the databases and search strategies, eligibility criteria, selection of studies, data extraction, and risk assessment methods, all adhering to the Preferred Reporting Items for Systematic Review and Meta-analysis of Diagnostic Test Accuracy Studies (PRISMA-DTA) guidelines. This is commendable as it enhances the transparency and replicability of the systematic review.	Thank you very much for the compliment.	
Page 6<br>*Lines 141-142*	Line 141-142; The title "Report characteristics" may not perfectly fit the content described in the paragraph. The text discusses specific criteria used to include or exclude studies for the systematic review, specifically the publication year and language, as well as the exclusion of study protocols. A section called 'types of studies' already discusses the studies to be included or excluded. The date range could be added to the databases searched and search strategies.	Removed the subheading "Report characteristics" and added the information "*on 9 February 2023 with no language restrictions"* under "Database and search strategies" instead.	Page 5<br>*Line 131*
Page 7<br>*Lines 160-161*	Line 160-161 – please check the heading 'risk of bias and acceptability' first sentence that followed 'risk of bias and applicability'. Can the authors confirm if acceptability and applicability are used interchangeably here?	Thank you for checking. This is a typographical error. Changed it already to "applicability" instead of "acceptability".	Page 8<br>*Line 194*

Page 7<br>*Lines152-153,* and<br><br>*Lines 164-165*	The authors should provide more clarity on resolving disagreements. While it's mentioned that a third reviewer would be consulted, the specific method or process (e.g., discussion, voting, senior author's decision) should be described.	Added phrases/words to provide more clarity on how disagreements were resolved for both the "Study Selection" and "Risk of bias and applicability" sections.	Page 8<br>*Lines 186-187* and *Line 199*
Page 5<br>*Lines 104-107*	The authors write, 'we also hand-searched the reference lists of relevant primary studies, systematic reviews, and the following journals: British Journal of Ophthalmology, American Journal of Ophthalmology, Ophthalmology and Retina, JAMA Ophthalmology, and Investigative Ophthalmology and Visual Science'. This search and the number of studies retrieved are not captured in the PRISMA flow chart or the findings. Can the authors clarify this?	The reason for not capturing this in the PRISMA flow diagram is that all the articles found using these were already included and accounted for in the electronic database search. Thus, we added the statement, *";and all these studies were already included or accounted for from the electronic database search, thus confirming the search strategy to be comprehensive."*	Page 5<br>*Lines 134-136*
Page 15<br>*Line 304*	While visual inspection of forest plots and HSROC plots are mentioned, the authors could have considered adding a quantitative assessment of heterogeneity using I-squared (I^2) statistics or Cochran's Q test might provide more accurate estimates of heterogeneity. Was there a reason this wasn't done?	I^2 statistic or Cochran's Q test were not used in this type of study for the following reasons stated below.<br><br>According to the PRISMA-DTA guidelines by Salameh et al. (2020)<br><br>*Methods for quantifying or describing heterogeneity in DTA systematic reviews used in intervention reviews cannot all be applied in DTA reviews. The I^2 statistic is not informative for DTA systematic reviews because it does not account for potential correlation between sensitivity and specificity, for example, owing to threshold effects. Multivariate and DTA specific I^2 statistics have been proposed to quantify heterogeneity, but they are not well established.*<br><br>Thus, visual inspection using forest plots and HSROC plots was used in our review as prescribed.	Page 16<br>*Line 379* (Exploring heterogeneity)
**Discussion and conclusion**			
Page 19	The Discussion section does an excellent job of summarizing the main findings and their implications and	Thank you very much for the compliment. Much appreciated!	

	provides context for these findings. The authors present their findings clearly, discussing the overall effectiveness of AI in screening for Referable Diabetic Retinopathy (RDR) in real-world settings. They also engage in hypothetical cohort modelling, which is a positive aspect, giving the reader a more intuitive understanding of the study's results.		
Page 19 *Lines 382-384*	The authors pointed out that their review provides evidence from prospective studies for the first time. They could elaborate on why this is significant and how their work contributes to or changes our current understanding of the field.	Thank you for this comment. We have elaborated on this in the "Implications for research" section on page 26, Lines 523-530.	
Page 19 *Lines 385-395*	The authors should provide more interpretation and discussion of their findings. For instance, why do they think the level of healthcare setting influenced diagnostic accuracy? Could factors like the complexity of cases, patient comorbidities, or healthcare workers' skill/experience level influence this result?	We added more interpretation and discussion of these findings.	Page 20 *Lines 463-471*<br><br>Page 21 Lines 482-486
**References**			
	Please review your reference list to ensure that it is complete and correct. If you have cited papers that have been retracted, please include the rationale for doing so in the manuscript text, or remove these references and replace them with relevant current references. Any changes to the reference list should be mentioned in the rebuttal letter that accompanies your revised manuscript. If you need to cite a retracted article, indicate the article's retracted status in the References list and also include a citation and full reference for the retraction notice.	Thank you! We added a couple of new references; thus, the numbering of citations (Vancouver format) has changed.<br><br>**Added references:**<br>**5.** Hazin R, Barazi MK, Summerfield M. Challenges to establishing nationwide diabetic retinopathy screening programs. Current Opinion in Ophthalmology. 2011;22: 174–179. https://doi.org/10.1097/icu.0b013e32834595e8<br><br>**8.** Li J-PO, Liu H, Ting DSJ, Jeon S, Chan RVPaul, Kim JE, et al. Digital technology, tele-medicine and artificial intelligence in ophthalmology: A global perspective. Progress in Retinal and Eye Research. 2020;82: 100900. https://doi.org/10.1016/j.preteyeres.2020.100900	Page 27 *Lines 636-638*<br><br>Page 28 *Lines 647-649*

		**11.** Arenas-Cavalli JT, Abarca I, Rojas-Contreras M, Bernuy F, Donoso R. Clinical validation of an artificial intelligence-based diabetic retinopathy screening tool for a national health system. Eye. 2022;36: 78–85. https://doi.org/10.1038/s41433-020-01366-0<br><br>**12.** Mokhashi N, Grachevskaya J, Cheng L, Yu D, Lu X, Zhang Y, et al. A comparison of artificial intelligence and human diabetic retinal image interpretation in an urban health system. Journal of Diabetes Science and Technology. 2021; 193229682199937. https://doi.org/10.1177/1932296821999370<br><br>**37.** EyePACS. Data Analysis. In: EyePACS [Internet]. [cited 17 Jul 2023]. Available: http://www.eyepacs.com/data-analysis<br><br>Added the word **"[video]"** in this reference:<br><br>**10.** London School of Hygiene & Tropical Medicine. Identifying a good screening test: sensitivity, specificity and coverage [video]. Future Learn. Available: https://www.futurelearn.com/info/courses/diabetic-eye-disease/0/steps/47640	Page 28<br>*Lines 655-657*<br><br>Page 28<br>*Lines 658-661*<br><br>Page 31<br>*Lines 742-743*<br><br>Page 28<br>*Lines 652-653*
**Supporting information**			
		**S2 Table. PRISMA-DTA**<br><br>For the supporting information (S2 Table), the pages for the PRISMA-DTA checklist will change depending on where the information will be in the final manuscript to be published. Kindly let us know if we can help provide these details (page numbers) before the publication. Thank you very much.	Page 32<br>*Lines 765*

--**END**--
