# Supplementary material for: Diagnostic test accuracy of artificial intelligence in screening for referable diabetic retinopathy in real-world settings: A systematic review and meta-analysis
Source: PLOS Glob Public Health. 2023 Sep 20;3(9):e0002160. doi: 10.1371/journal.pgph.0002160 (PMC10511145; doi:10.1371/journal.pgph.0002160)
Supplement: Attachment — Submitted filename: Reviewer report.docx [file pgph.0002160.s003.docx]

**Abstract**

The abstract is well-written and provides information about the study's methods, results, and interpretation. It presents the aim, methodology, key findings, and conclusions succinctly. However, here are a few suggestions to improve the clarity and readability:

- Be more specific with terms like 'moderate increase' and 'minimal decrease'. If possible, provide exact numbers or percentages to give the reader a clear understanding of the changes.
- The conclusion might benefit from more explicit statements about the implications of the findings. You've mentioned that AI is effective in screening for RDR, but you could discuss how this impacts current practice or what the next steps could be.
- Line 35: The abbreviation SROC, did you want to write HSROC?

**Introduction**

The introduction is detailed and provides a good overview of the topic, clearly establishing the importance and urgency of the research. The problem is well-defined, and the literature gap that this study intends to address is well-articulated. However, a few areas could be improved:

- A brief historical context could help to put this research’s importance into perspective. For instance, what has been the trajectory of DR treatments? What has been the impact of AI in healthcare generally?
- Line 80-81: Ensure all the information presented has appropriate and recent references. For example, the statement "In recent years, retrospective validation studies have shown AI to have high diagnostic accuracy in detecting DR..." needs a reference citation.

**Methods**

The Methods section of the systematic review appears to be largely comprehensive and transparent in its description of the processes and criteria used for the review. It carefully outlines the databases and search strategies, eligibility criteria, selection of studies, data extraction, and risk assessment methods, all adhering to the Preferred Reporting Items for Systematic Review and Meta-analysis of Diagnostic Test Accuracy Studies (PRISMA-DTA) guidelines. This is commendable as it enhances the transparency and replicability of the systematic review.

- Line 141-142; The title "Report characteristics" may not perfectly fit the content described in the paragraph. The text discusses specific criteria used to include or exclude studies for the systematic review, specifically the publication year and language, as well as the exclusion of study protocols. A section called ‘types of studies’ already discusses the studies to be included or excluded. The date range could be added to the databases searched and search strategies.
- Line 160-161 – please check the heading ‘risk of bias and acceptability’ first sentence that followed ‘risk of bias and applicability’. Can the authors confirm if acceptability and applicability are used interchangeably here?
- The authors should provide more clarity on resolving disagreements. While it's mentioned that a third reviewer would be consulted, the specific method or process (e.g., discussion, voting, senior author's decision) should be described.
- The authors write, ‘we also hand-searched the reference lists of relevant primary studies, systematic reviews, and the following journals: British Journal of Ophthalmology, American Journal of Ophthalmology, Ophthalmology and Retina, JAMA Ophthalmology, and Investigative Ophthalmology and Visual Science’. This search and the number of studies retrieved are not captured in the PRISMA flow chart or the findings. Can the authors clarify this?
- While visual inspection of forest plots and HSROC plots are mentioned, the authors could have considered adding a quantitative assessment of heterogeneity using I-squared (I²) statistics or Cochran's Q test might provide more accurate estimates of heterogeneity. Was there a reason this wasn’t done?

Discussion and conclusion

The Discussion section does an excellent job of summarizing the main findings and their implications and provides context for these findings. The authors present their findings clearly, discussing the overall effectiveness of AI in screening for Referable Diabetic Retinopathy (RDR) in real-world settings. They also engage in hypothetical cohort modelling, which is a positive aspect, giving the reader a more intuitive understanding of the study's results.

- The authors pointed out that their review provides evidence from prospective studies for the first time. They could elaborate on why this is significant and how their work contributes to or changes our current understanding of the field.
- The authors should provide more interpretation and discussion of their findings. For instance, why do they think the level of healthcare setting influenced diagnostic accuracy? Could factors like the complexity of cases, patient comorbidities, or healthcare workers' skill/experience level influence this result?
